# Meta‐analysis shows both congruence and complementarity of DNA and eDNA metabarcoding to traditional methods for biological community assessment

**DOI:** 10.1111/mec.16364

**Published:** 2022-02-02

**Authors:** François Keck, Rosetta C. Blackman, Raphael Bossart, Jeanine Brantschen, Marjorie Couton, Samuel Hürlemann, Dominik Kirschner, Nadine Locher, Heng Zhang, Florian Altermatt

**Affiliations:** ^1^ Department of Aquatic Ecology Eawag: Swiss Federal Institute of Aquatic Science and Technology Dübendorf Switzerland; ^2^ 27217 Department of Evolutionary Biology and Environmental Studies University of Zurich Zürich Switzerland; ^3^ 27217 Research Priority Programme Global Change and Biodiversity (URPP‐GCB) University of Zurich Zürich Switzerland; ^4^ Landscape Ecology Institute of Terrestrial Ecosystems Department of Environmental System Science ETH Zürich Zürich Switzerland; ^5^ Landscape Ecology, Land Change Science Swiss Federal Institute for Forest, Snow and Landscape Research WSL Birmensdorf Switzerland

**Keywords:** diversity assessment, DNA, fish, invertebrates, meta‐analysis, metabarcoding, microorganisms

## Abstract

DNA metabarcoding is increasingly used for the assessment of aquatic communities, and numerous studies have investigated the consistency of this technique with traditional morpho‐taxonomic approaches. These individual studies have used DNA metabarcoding to assess diversity and community structure of aquatic organisms both in marine and freshwater systems globally over the last decade. However, a systematic analysis of the comparability and effectiveness of DNA‐based community assessment across all of these studies has hitherto been lacking. Here, we performed the first meta‐analysis of available studies comparing traditional methods and DNA metabarcoding to measure and assess biological diversity of key aquatic groups, including plankton, microphytobentos, macroinvertebrates, and fish. Across 215 data sets, we found that DNA metabarcoding provides richness estimates that are globally consistent to those obtained using traditional methods, both at local and regional scale. DNA metabarcoding also generates species inventories that are highly congruent with traditional methods for fish. Contrastingly, species inventories of plankton, microphytobenthos and macroinvertebrates obtained by DNA metabarcoding showed pronounced differences to traditional methods, missing some taxa but at the same time detecting otherwise overseen diversity. The method is generally sufficiently advanced to study the composition of fish communities and replace more invasive traditional methods. For smaller organisms, like macroinvertebrates, plankton and microphytobenthos, DNA metabarcoding may continue to give complementary rather than identical estimates compared to traditional approaches. Systematic and comparable data collection will increase the understanding of different aspects of this complementarity, and increase the effectiveness of the method and adequate interpretation of the results.

## 1 INTRODUCTION

Assessment of biological assemblages is key to almost every study in ecology (Hampton et al., 2013), and managing and preserving ecosystems requires a global effort to regularly monitor the composition and diversity of their biological communities (Jørgensen et al., 2010). In aquatic ecosystems, routine biological assessment has a long history and a wide range of groups of organisms (such as diatoms, aquatic plants, invertebrates, and fish) are monitored to evaluate the state and change of aquatic environments over time, and to assess different types of human‐induced pressures and impairments (Barbour et al., 1999; Borja et al., 2000; Hering et al., 2018). In order to use these organisms in large ecological monitoring programmes, a variety of methods based on capture of individuals (such as biofilm collection, sampling by net, or electro‐fishing) have been developed and standardized. However, the taxonomic identification remains achieved with morphological criteria, a time consuming task that is further prone to errors and achieved at family or genus taxonomic levels for some groups (Haase et al., 2006; Mandelik et al., 2010).

Technological advances in high throughput DNA sequencing and data analyses are currently revolutionizing biodiversity sciences, and are providing a novel approach to characterize biodiversity of whole communities by using the DNA of organisms for their taxonomic identification (Hering et al., 2018; Leese et al., 2016). Thereby, metabarcoding can either be based on DNA extracted from bulk samples (i.e., pools of organisms) or from environmental samples (eDNA), with subsequent amplification of a specific gene region in target taxonomic groups using a dedicated primer pair (Deiner et al., 2017; Leese et al., 2016; Pawlowski et al., 2020b; Taberlet et al., 2012). For the purpose of this study we will refer to DNA metabarcoding of eDNA and bulk samples as “DNA metabarcoding” hereafter. DNA metabarcoding is transforming how plants and animals are surveyed (Deiner et al., 2017) by solving several constraints associated with traditional methods and is presented as being cheaper, faster, more sensitive, and easily scalable for routine monitoring programmes (Altermatt et al., 2020; Hering et al., 2018; Leese et al., 2016).

Given the stakes behind the implementation of DNA metabarcoding for academics and stakeholders (Bruce et al., 2021; Pawlowski et al., 2020a, 2020b), the last decade has seen a growing number of studies testing DNA metabarcoding effectiveness in quantifying biodiversity and detecting species present in the environment. It is needed for two main reasons. First, to validate the concept and the protocols against methods, which have been applied for decades and whose performance and limitations are well documented. Second, to ensure the continuity of long‐term traditional monitoring time series. Accordingly, many case‐ or site‐specific studies have attempted to estimate the congruence between the two approaches, that is, comparing the diversity of organisms assessed with DNA metabarcoding to assessments with traditional methods (e.g., Abad et al., 2016; Cahill et al., 2018; Fernández et al., 2018; Hänfling et al., 2016; Leese et al., 2021; Li et al., 2019a, 2019b; Mächler et al., 2019; Vasselon et al., 2017). These studies have not only been conducted on a broad range of aquatic ecosystems, individual study sites and organismal groups, but are also reporting a wide range of comparability versus divergence to classically assessed community data. For example, individual studies often report that metabarcoding detects a substantial proportion of species identified on morphological criteria. However, the variability between studies is high, and often, a significant fraction of diversity is still only detected by one or the other approach (e.g., Apothéloz‐Perret‐Gentil et al., 2017; Aylagas et al., 2016; Kelly et al., 2017; Polanco Fernández et al., 2021; Vasselon et al., 2017). General knowledge on variability (or consistency) across studies is needed to inform about the specificities and limitations of each approach and to thus allow us to define the best strategies for current and future biomonitoring programs (Hering et al., 2018). To our knowledge, there has been no attempt to comprehensively review and analyse the available information in a systematic and quantitative way.

Here, we conducted the first systematic meta‐analysis of the available studies that have compared the performances of DNA metabarcoding with traditional methods to estimate the diversity of a variety of organisms in aquatic ecosystems, both marine and freshwater. Specifically, we investigated taxonomic richness (i.e., the number of taxa detected) and taxonomic composition (i.e., the identity of the taxa detected). These two elements are major components of biodiversity and their role on the stability and functioning of ecosystems has been clearly demonstrated in the past (Cardinale et al., 2006; Pennekamp et al., 2018). We chose not to include abundance‐related parameters in the analysis since this aspect is still controversial in metabarcoding studies (Lamb et al., 2019). We first evaluated which method detects the highest taxonomic diversity. Second, we investigated if and to what extent these methods are congruent in the taxa detected. Finally, we examined the spatial scale (local versus regional) of this congruence and if the congruence between traditional methods and metabarcoding is dependent on the group of targeted organisms (plankton/microphytobenthos, macroinvertebrates, and fish). By aggregating the large body of literature available and analysing them through a straightforward meta‐analysis, we aim to end speculation as to the abilities of both methods for monitoring aquatic systems.

## 2 MATERIALS AND METHODS

### 2.1 Data collection

We conducted a systematic and comprehensive meta‐analysis on all available, published studies following a set of formal criteria. Studies reporting comparisons between DNA metabarcoding and traditional methods to assess biological diversity in aquatic ecosystems (marine and freshwater) were searched using the online database Web of Science Core Collection (Clarivate Analytics) on 25 February 2021 (see Supporting Information S1 for the complete query used for the search). The combination of keywords used as search terms was chosen to be as specific as possible to include any studies comparing DNA metabarcoding to a traditional method (including both capture and identification) in aquatic ecosystems. Additionally, the search was limited to articles published between 2010 and 2021, since no study using metabarcoding on our targeted groups was expected prior to 2010 as the methods and apparatus needed were unavailable. The initial search was complemented with a manual inspection of all articles published in *Metabarcoding and Metagenomics* and *Environmental DNA*, two new journals specialised in metabarcoding and environmental DNA (eDNA) studies, but whose publications were not yet indexed by Web of Science.

The initial search output was then carefully screened by manually checking the title, abstract and, when necessary, the complete content of the articles and their supplementary material, in order to retain only those articles that met our inclusion criteria. To be included in our analysis, studies had to report a comparison between DNA metabarcoding (on bulk or eDNA samples) and one or several traditional methods to assess biological diversity in aquatic ecosystems (marine or freshwater). The sample types included water samples, biofilm samples, as well as bulk‐metabarcoding. The diversity estimated by the two approaches had to be reported using quantitative values expressed as taxonomic richness (i.e., the number of detected taxa). When this information was not directly available, but authors provided taxonomic lists obtained with metabarcoding and traditional methods, we were able to estimate the values of diversity needed for the analyses and included the studies as well. We included only studies using DNA metabarcoding to assess taxonomic diversity at the community level and therefore excluded studies using conventional PCR, qPCR, ddPCR, or barcoding for single species detection. Studies using artificially assembled communities (e.g., mock communities, aquarium) were also excluded. Finally, we excluded studies focusing on bacteria and fungi for which communities are rarely assessed using traditional methods.

### 2.2 Data extraction

For each comparison between a traditional method and DNA metabarcoding, the measures of diversity (richness) were extracted from the articles and the supplementary materials published by the authors at two different levels: local alpha diversity (i.e., the average diversity per site) and regional gamma diversity (i.e., the total diversity across sites). For each study, data were extracted at the lowest possible taxonomic level that was common to both approaches (generally species, genus or family level).

**FIGURE 1:**
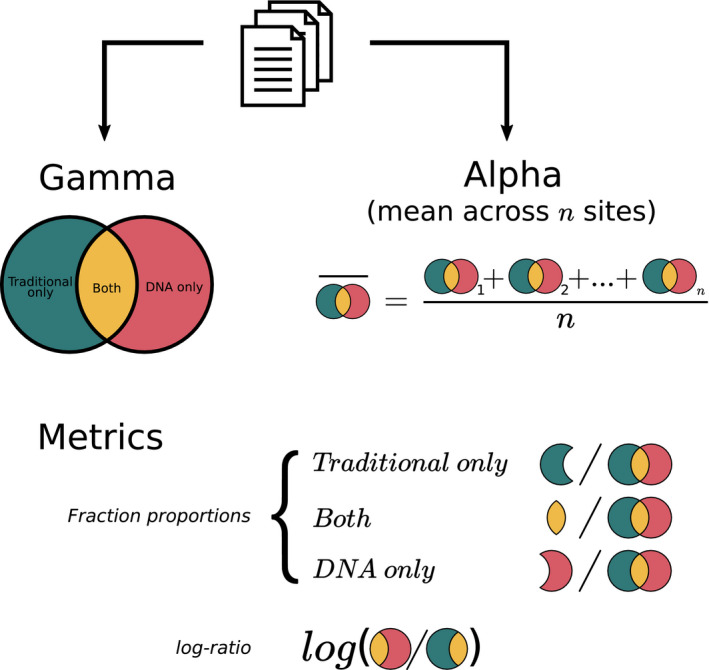
Study workflow. Gamma diversity (i.e., regional richness) and alpha diversity (i.e., local richness) values were extracted for different taxonomic groups from 99 studies. For each type of diversity, the relative fraction of taxa detected by the traditional method only (green), by DNA metabarcoding only (red) and by both methods (yellow) were compared. The log‐ratio between the total diversity detected by DNA metabarcoding and the total diversity detected by the traditional method was also assessed [Color figure can be viewed at wileyonlinelibrary.com]

When taxonomic diversity (here: richness) is assessed with two different approaches (here: traditional and DNA metabarcoding), the total number of detected taxa can be decomposed into three subsets: (1) the subset of taxa detected only by the traditional method, (2) the subset of taxa detected only by DNA metabarcoding, and (3) the subset of taxa detected by both methods, (i.e., the intersection subset; Figure 1).

For gamma diversity, measures of these three subsets are often reported in publications in the main text or in figures (e.g., Venn diagrams or stacked bar plots) and were directly extracted for each comparison. In contrast, the subsets of taxa detected only by one or the other method are rarely reported at the level/resolution of each site, but are often available in an integrated manner only. Therefore, for each method, we computed an average of alpha diversity (local richness) across all sites. We did the same for the intersection fraction (i.e., the subset of taxa detected by both methods). The intersection fraction for alpha diversity is rarely reported, but could be estimated when the articles provided detailed lists of taxa detected by the two methods. Finally, we estimated the “traditional only” fraction and the “DNA only” fraction by subtracting the intersection fraction from the traditional and DNA total alpha diversity.

Along with the measures of diversity, we also extracted key variables of interest, including article metadata, information on the study designs (sampled habitat, targeted taxonomic group, taxonomic resolution), and methodological details about the metabarcoding approach (markers, primers, technologies). The complete list of extracted data is provided as Table S1. To simplify the analyses and ease the reading of the results, the organisms targeted in the included studies were grouped into four categories: plankton and microphytobenthos (including diatoms, zooplankton, phytoplankton, cyanobacteria, and protists), macroinvertebrates, fish, and “others” (e.g., corals, macrophytes, amphibians); the latter only containing a small number of studies.

### 2.3 Statistical analyses

To assess whether one approach detected more diversity (richness) than the other, we used the log‐ratio *ln(A*/*B)*, where, for each method comparison, *A* is the total diversity detected by DNA metabarcoding and *B* is the total diversity detected by the traditional method (Figure 1). The log‐ratio is a widely used effect size measure in ecological meta‐analyses to summarize the magnitude and direction of multiple research outcomes (Hedges et al., 1999). The log‐ratio value is positive when *A* is greater than *B*, negative when *B* is greater than *A*, and zero when *A* and *B* are equal (i.e., the two methods estimate the same diversity). To test which method detected the highest diversity, we used linear mixed models with the study block as random effect (intercept). Two models were fitted: an intercept‐only model to address the mean log‐ratio, and one model including the group of organisms as an independent variable to test possible effects of the type of taxa on the log‐ratio.

To analyse the congruence between traditional methods and DNA metabarcoding, we compared the three fractions (traditional only, DNA metabarcoding only and the intersection fraction, see above and Figure 1). Fractions were standardised by turning them into proportions with values ranging from zero to one to allow cross‐study comparisons. To test differences between fractions within and across groups of taxa, we used a beta‐regression mixed model (Ferrari & Cribari‐Neto, 2004), including the taxonomic groups (plankton and microphytobenthos, macroinvertebrates, and fish) and the types of fraction (traditional only, DNA only, and both) as independent variables. The beta‐regression is based on the assumption that the response variable is beta‐distributed, and is particularly well adapted to study proportions. Two models were fitted separately for gamma and alpha diversity and two levels of nested random effects (intercepts) were specified (studies and comparisons) for these models. Proportion values were compressed using the method of Smithson and Verkuilen (2006) to avoid true zeros and ones in the beta‐regression. Due to a major lack of data in the other taxonomic levels and the large class imbalance, the model was fitted only for the comparisons made at species level (i.e., the level for which we had the most data available). Beta‐regression models were completed with post hoc pairwise comparisons among groups based on estimated marginal means with a Tukey's procedure to control for family‐wise error rate.

We performed all the statistical analyses with R 4.0.3 software (R Core Team, 2020). Generalized linear mixed models were fitted using the glmmTMB package (Brooks et al., 2017) and the emmeans package was used to perform post hoc pairwise comparisons (Lenth, 2021).

## 3 RESULTS

From the 1,217 studies initially identified by the literature search, many focused on single species assays or bacteria only, and were thus excluded. In total, 99 studies met our inclusion criteria and were used in the analyses (Table 1, Supporting Information S2). From these, we extracted a total number of 215 comparisons (some articles presented several comparisons using different primers or different taxonomic groups assessed separately) of diversity measurements between a traditional method and DNA metabarcoding. Most of these comparisons were presented at regional level, that is, gamma diversity (188 comparisons). A relatively large number of studies reported data for each approach at site level (120 comparisons), while the intersection fraction between the traditional and DNA methods could be extracted for 88 comparisons. All the extracted data are available (Keck et al., 2021).

**TABLE 1:** List of articles included in the meta‐analysis sorted by group of organisms

Group of organisms	Articles included
Plankton and microphytobenthos	Abad et al. (2016), Apothéloz‐Perret‐Gentil et al. (2017, 2020), Bachy et al. (2013), Bailet et al. (2020), Clarke et al. (2017), Djurhuus et al. (2018), Dzhembekova et al. (2020), Eiler et al. (2013), Harvey et al. (2017), Hirai et al. (2015), Huang et al. (2020), Huo et al. (2020), Kang et al. (2021), Kermarrec et al. (2014), Kim et al. (2019), Kim et al. (2020), Li et al. (2019b), Liu et al. (2017), Minerovic et al. (2020), Mora et al. (2019), Nunes et al. (2019), Pérez‐Burillo et al. (2020), Pujari et al. (2019), Rivera et al. (2018b), Rivera et al. (2018a), Schroeder et al. (2020), Semmouri et al. (2021), Vasselon et al. (2017), Visco et al. (2015), Xiao et al. (2014), Yang et al. (2017), Zimmermann et al. (2015)
Macroinvertebrates	Aylagas et al. (2016), Aylagas et al. (2018), Azevedo et al. (2020), Borrell et al. (2017), Cahill et al. (2018), Cowart et al. (2015), Elbrecht et al. (2017), Emilson et al. (2017), Erdozain et al. (2019), Fernández et al. (2018), Fernández et al. (2019), Haenel et al. (2017), Harper et al. (2020), Kelly et al. (2017), Krol et al. (2019), Kuntke et al. (2020), Laini et al. (2020), Leese et al. (2021), Lejzerowicz et al. (2015), Lobo et al. (2017), Mächler et al. (2019), Marshall and Stepien (2020), Martins et al. (2019), Obst et al. (2020), Rivera et al. (2021), Serrana et al. (2018), Serrana et al. (2019), Steyaert et al. (2020), Sun et al. (2019), Uchida et al. (2020), Vivien et al. (2019)
Fish	Afzali et al. (2021), Aglieri et al. (2020), Berger et al. (2020), Bleijswijk et al. (2020), Boivin‐Delisle et al. (2021), Bylemans et al. (2018), Cilleros et al. (2019), Closek et al. (2019), Collins et al. (2019), Doble et al. (2020), Evans et al. (2017), Fujii et al. (2019), Goutte et al. (2020), Hänfling et al. (2016), Hayami et al. (2020), McClenaghan et al. (2020), McDevitt et al. (2019), Nguyen et al. (2020), Oka et al. (2021), Olds et al. (2016), Polanco Fernández et al. (2021), Pont et al. (2018), Port et al. (2016), Sakata et al. (2021), Sard et al. (2019), Shaw et al. (2016), Snyder and Stepien (2020), Thomsen et al. (2012), Valentini et al. (2016)
Others (including multiple groups)	Alsos et al. (2018), Deagle et al. (2018), Gran‐Stadniczeñko et al. (2017), Leduc et al. (2019), Nichols and Marko (2019), Shackleton et al. (2019), Valentini et al. (2016)

**FIGURE 2:**
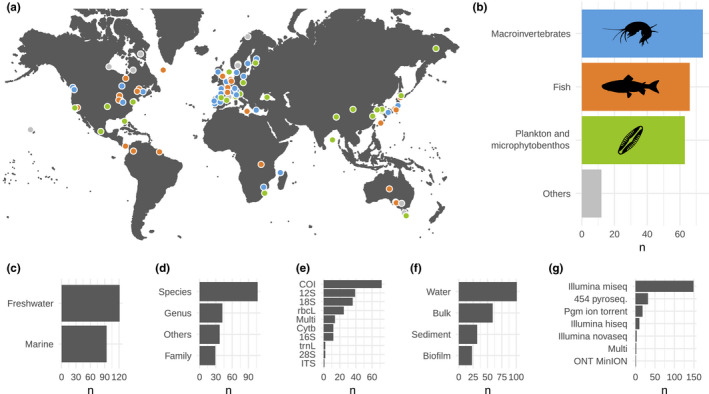
Overview of the different comparisons extracted from the studies included in the meta‐analysis. (a) Geographic location of the comparisons. Colours indicate the group of organisms used (plankton and microphytobenthos in green, macroinvertebrates in blue, fish in orange and other types in light grey). (b) Number of comparisons (n) across the different groups of organisms. (c–g) Number of comparisons (n) across biomes (marine includes brackish waters), taxonomic levels of identification, genetic markers, origins of DNA and sequencing technologies. The “multi” category includes comparisons combining several other categories

The data included several continents and climatic regions. However, they are strongly spatially aggregated and most studies are focused on Europe and North America (Figure 2a). The data set covers a large variety of functional and taxonomic groups that range from microbial species to fish (Figure 2b), representing an important variation in body size or trophic position. Both marine (44% of comparisons) and freshwater ecosystems (56%) are represented (Figure 2c), with data available for numerous types of aquatic environments, ranging from small streams and ponds to oceans (Figure 2a). Most comparisons were made and reported by the authors at species level (49.8% of comparisons), although a significant number of studies investigated diversity at higher taxonomic levels (Figure 2d). The variety of studied organisms and habitats is reflected by the diversity of genetic markers (9 markers, 64 different primer pairs) and source of DNA used by the authors (Figure 2e–f). Finally, several sequencing technologies are represented in the data set, with Illumina MiSeq being the most commonly used technology over the studied period (69.3% of comparisons; Figure 2g). Detailed distributions of the recorded observations are shown in Figure S1 and S2.

**FIGURE 3:**
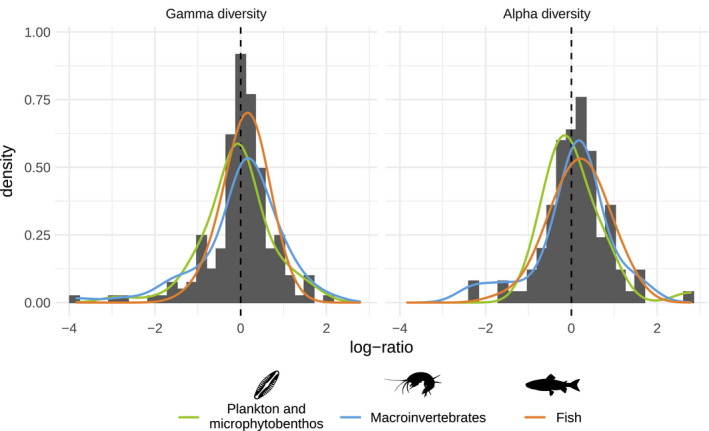
Histograms of the log‐ratio between the total diversity detected by DNA metabarcoding and the total diversity detected by the traditional method. The left panel shows gamma diversity (i.e., regional richness) and the right panel alpha diversity (i.e. mean local richness). Density estimates (kernel bandwidth = 0.25) for each group of organisms are represented as coloured overlays [Color figure can be viewed at wileyonlinelibrary.com]

At regional level (gamma diversity), we found that there was no one approach that detected higher richness than the other (Figure 3). We measured an average log‐ratio between DNA metabarcoding and the traditional method of –0.010 (sd = 0.764) which was not found to be significantly different from zero by the statistical model (intercept = –0.016, Z‐value = –0.187, *p* = .851) and the effect of the group of organisms was not found significant (Wald Chi2 = 2.078, df =3, *p* =.556). At local scale (alpha diversity), the average log‐ratio was 0.093 (sd = 0.73) and was found marginally‐significant in the model (intercept = 0.167, Z‐value = 1.979, *p* = .048), suggesting that DNA metabarcoding could detect more taxa, on average, than traditional methods. Similarly to the gamma diversity, the effect of the group of organisms was not found significant for alpha diversity (Wald Chi2 = 0.486, df = 3, *p* = .922). Details on the estimated terms of the models are provided as (Table S2).

**FIGURE 4:**
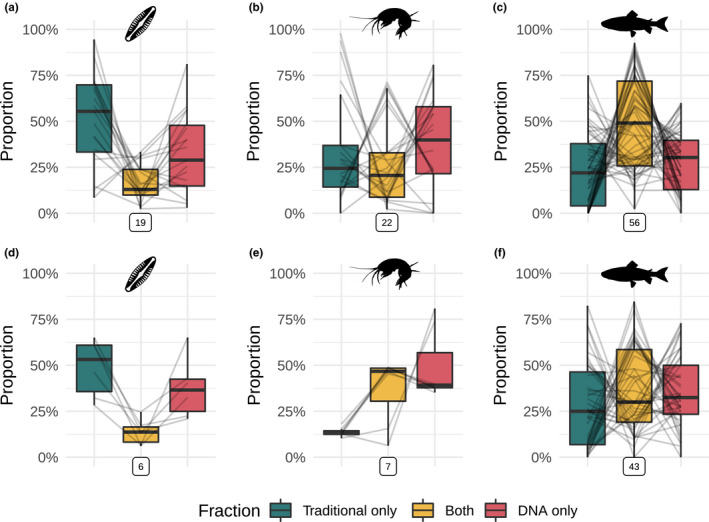
Relative fraction of diversity detected by the traditional method only, by DNA metabarcoding only and by both methods. Data are presented for different groups of organisms identified at species level only. Boxplots show medians, first and third quartiles, and full ranges (limited to 1.5 × interquartile range). Grey lines connect values from the same comparison. Framed numbers below each panel indicate the number of comparisons represented. (a–c) Gamma diversity for plankton and microphytobenthos, macroinvertebrates and fish. (d–f) Alpha diversity for plankton and microphytobenthos, macroinvertebrates and fish [Color figure can be viewed at wileyonlinelibrary.com]

Overall, we found that the proportion of diversity detected strongly varied accross fractions (traditional only, DNA only, or both methods) and groups of organisms (Figure 4 for species, see Figures S3 and S4 for the other taxonomic levels). This was confirmed both for alpha and gamma diversity by the analyses of deviance of the beta‐regression models (interaction term *p* = .007 and < .001 respectively, Table S3). Further, we found that the fractions of gamma diversity detected only by the traditional method and only by DNA metabarcoding were significantly lower than the fraction detected by both methods for fish (Table S4). This trend is inverted for plankton and microphytobenthos (Figure 4a), although only the “traditional‐only” fraction was significantly higher than the “both” fraction (Table S4). Macroinvertebrates are intermediate, with none of the fraction being significantly higher than the others (Figure 4b and Table S4). Very similar trends were observed for alpha diversity (Figure 4d–f), but none of the pairwise tests showed significant differences (Table S5), probably because of a lack of statistical power.

We also observed a high variability in the proportion of species found with both methods, ranging from 2% (macroinvertebrates) to 93% (fish). This variability was also important within taxonomic groups (e.g., ranging from 2% to 71% in macroinvertebrates, Figure 4).

Besides the group of organisms, we also recorded and explored the effects of other factors like the origin of DNA or the difference between marine and freshwater systems. The effects of these factors and especially the combined effects of these factors with the effect of the group of organisms are only visually presented (Figures S5–S8), but were not formally tested because of a lack of data, class imbalance, and the high multicollinearity among variables (e.g., between the groups of organisms and the origin of DNA). In addition, we extracted several other variables specific to each study that could have an influence on the comparison between DNA metabarcoding and traditional methods (Figures S2). This included the total number of reads available (ranging from 2 × 10^4^ to 2.7 × 10^8^), the number of sites (ranging from 1 to 164) and samples (ranging from 1 to 373) studied, or the year of publication of the study (ranging from 2012 to 2021, possibly conditioning the sequencing and bioinformatics techniques used and associated to the completeness of the reference databases used at the time). All these variables are of possible further interest, but can also be sources of variation, probably to generate statistical noise, which are controlled for in our analyses by including the study block as random effect in all models.

## 4 DISCUSSION

Since the initial proposal to use metabarcoding to simultaneously identify all organisms in the environment (Ficetola et al., 2008; Jerde et al., 2011; Taberlet et al., 2012, see also Pawlowski et al., 2020b for a recent commentary on this), many scientists are intrigued by or have questioned its comparability with the traditional methods used for biodiversity inventories. In order to synthesise and provide a quantitative basis of the current state of knowledge on this issue, we collected and analysed all available data from a large corpus of publications comparing traditional methods with DNA metabarcoding.

At regional level (gamma diversity), we found that there was no one approach that detected more diversity (richness) than the other. This result indicates that the regional richness estimated by metabarcoding matches well with the traditional methods, hence confirming the potential of this approach for biodiversity assessment in aquatic ecosystems. This is, however, an overall trend across all studies, while the distribution of log‐ratio (Figure 3a) shows a wide range of situations, with some rare cases where the absolute log‐ratio reaches 2 (i.e., one method detected seven times more taxa at the regional scale than the other). Unlike gamma diversity, the log‐ratio was found to be significantly higher than zero for alpha diversity, suggesting that at site level, DNA metabarcoding detects more diversity than its traditional counterparts. This result should be interpreted with caution, especially in view of the small effect size reported (on average metabarcoding detected 1.1 times more taxa than the traditional methods), but is in line with a recent meta‐analysis showing that the probability of species detection is higher with eDNA than with traditional methods (Fediajevaite et al., 2021).

Traditional methods and their respective efficacy will probably remain stable in the future as those methods have been established, optimised and standardized over decades. Contrastingly, the techniques behind DNA metabarcoding are continuously refined and the approach has probably not yet reached its full potential in its ability to detect taxonomic diversity (Keck et al., 2017). In particular, the reference databases used to evaluate taxon‐occurrences are far from complete (Weigand et al., 2019). This variability and historic development of the metabarcoding approach can also be seen in our data by the large number of approaches and protocols used across the studies reviewed, resulting in a large variation in sequencing depth, number of investigated sites, and replicates. Such variation may be inherent and typical for a young field of research and partially obscure the differences between the two approaches, but this is precisely the aim of a meta‐analysis to combine results from heterogeneous research while controlling for possible variations across studies. We may expect that progress and standardization in DNA sampling techniques, sequencing technologies, bioinformatics processing, and reference database coverage will improve the capacity of DNA metabarcoding to estimate diversity. This margin of progress may suggest that metabarcoding could widen the gap with traditional approaches in terms of its ability to measure taxonomic richness in the future, as the former is expected to improve further.

Our analyses did not demonstrate a significant effect of the type of organism on the log‐ratio between the number of taxa detected by DNA and traditional methods. This is surprising and a bit unexpected, as, especially for macroinvertebrates, there have been extensive debates in the literature about the suitability of (e)DNA metabarcoding (Blackman et al., 2019; Elbrecht et al., 2017). The large variation observed in the log‐ratio across studies, however, suggests that the accuracy and overlap depends also on the combination of many other factors, which can not be modelled here due to a lack of data. Besides the group of organisms, the context of the study and the methods used can also have drastic effects on the relative estimates of biodiversity provided by DNA metabarcoding and traditional methods. For instance, eDNA in lentic environments may persist longer than in lotic or marine systems (Collins et al., 2018; Dejean et al., 2011), which implies that metabarcoding data might have a better congruence with traditional methods due to a better temporal congruence. Similarly, eDNA from lotic and marine systems is influenced by transportation from flow (Deiner & Altermatt, 2014) and ocean currents (Harrison et al., 2019), meaning that the DNA/eDNA does not represent the same spatial scale as a traditional sample from the same environment. Therefore, eDNA samples will generally reflect different spatio‐temporal scales (Civade et al., 2016; Deiner et al., 2016) compared to traditional point sampling, such as kick‐nets (Mächler et al., 2019). Thus, the large diversity of habitats and sampling strategies in our data set can possibly explain the large variation in log‐ratio values. We also note that there is a major difference across the different taxonomic groups with respect to general species richness, specificity of primers used, completeness of reference databases, sequencing depths and taxonomic scales (see also Table S2). This creates possible biases that will also affect the log‐ratios, and comparability of biodiversity detected across organismal groups. For example, fish are a relatively well‐defined and small taxonomic group. They are generally assessed with highly specific primers, and mostly resolved to the species level. In contrast, macroinvertebrates are a highly diverse, phylogenetically poorly defined and species‐rich group. Moreover, they are usually assessed with primers amplifying also many nontarget organisms, and their assignment is often only possible to the genus or family level. Thus, it is not surprising that the former, fish, are better covered by metabarcoding methods with results matching more closely traditional approaches. However, major improvements are yet to come, especially for the less efficient groups, with the production of reference sequences, the increase in sequencing depths and the development of more specific primers. Our analysis should thus not only be seen as an assessment of past studies, but can also reveal in which fields and organismal groups further research should be conducted to achieve a better effectiveness of the metabarcoding approaches.

Although DNA and traditional methods estimated the same number of taxa (diversity) on average, our results suggest that they often do not count the same taxa. This is shown by the fraction of species detected by both methods which is particularly low for plankton, microphytobenthos and macroinvertebrates. This result has important implications, because in addition to taxonomic richness, taxonomic composition is an essential element of biodiversity. Ecologists are interested in species identities in biological assemblages because they are rich in information about environmental quality and ecosystem functioning. Additionally, being able to identify taxa is important to monitor rare and endangered species or to detect invasive species. It is worth noting that, in this study, the correspondence between metabarcoding and traditional taxonomic lists is only based on the exact match between taxa names. The taxonomic and phylogenetic distances between the methods are likely to be less significant, and can be used to optimize the calculation of ecological quality indices (Keck et al., 2018). Such metrics, however, are rarely reported and could not be evaluated.

For plankton and microphytobenthos, which are mainly represented by planktonic protists and benthic diatoms, the discrepancies between traditional methods and DNA metabarcoding can be attributed to the respective flaws of these two approaches, which are well documented. Traditional approaches that rely on microscopic morphological characters for species identification are known to be a source of errors. In particular, several species and species complexes, which have been initially separated using molecular methods, are difficult to identify on the sole basis of light microscopy (Jahn et al., 2017; Pinseel et al., 2017). Contrastingly, the DNA metabarcoding approach is also limited in recovering diversity unveiled by traditional methods. Firstly, the short DNA fragment used as a genetic barcode can be insufficient (in size and/or variability) to separate morphotaxonomic species (Apothéloz‐Perret‐Gentil et al., 2017). Secondly, reference databases are still incomplete in terms of the diversity found in microeukaryotic communities, and many environmental sequences cannot be accurately classified at species level (Lindeque et al., 2013; Weigand et al., 2019). In this respect, coordinated efforts to build extensive reference databases for protists and other microeukaryotic species (e.g., Guillou et al., 2013; Rimet et al., 2019) are particularly important and are expected to improve metabarcoding performances in the future.

As in the case of planktonic and microphytobenthic organisms, the fraction of macroinvertebrate species detected by both methods (i.e., overlap in identity of organisms detected) is remarkably low. Again, the discrepancies between the lists of taxa produced using DNA metabarcoding and traditional morphological analysis can be explained by the inherent biases of both methods, as described above (Lobo et al., 2017). Furthermore, it is important to mention that the taxonomic extent targeted by the two methods can be very different. The generic primers used for macroinvertebrate metabarcoding are often rather unspecific and target a diversity of organisms (based on their phylogenetic and thus primer‐binding site similarity), which is often overlooked by the operators performing traditional identification (Elbrecht et al., 2017). The recent development of specific primers for macroinvertebrate communities could help address this issue (Leese et al., 2021), but probably will not completely resolve it, as a polyphyletic group such as “macroinvertebrates”, solely defined by their size, may never be adequately captured by genetic methods without also including closely related but smaller organisms. Additionally, the large variance observed in the fraction detected by both methods can be explained by the fact that our data set includes both environmental DNA and bulk samples for macroinvertebrates. Recent studies have shown that environmental DNA includes a larger diversity of taxa than bulk samples, but many are nontargeted and do not match the taxa identified by traditional methods (Gleason et al., 2020; Macher et al., 2018).

For fish, the observed trend is clearly reversed to what we see with plankton, microphytobenthos, and macroinvertebrates. The fraction of fish species detected by both methods indicates that the concordance is good between DNA metabarcoding and the traditional approaches. This result is in line with conclusions from individual studies (e.g., Hänfling et al., 2016; Li et al., 2019a, 2019b; Pont et al., 2018; Valentini et al., 2016) and with a recent synthesis by McElroy et al. (2020). The good match between taxonomic lists generated by the two approaches can be explained by the limited regional diversity of fish communities investigated in the available studies. Compared to plankton, microphytobenthos, and macroinvertebrates, regional fish faunas are often well documented, and reference databases are extensive and can be easily completed by sequencing new individuals (Valentini et al., 2016). However, the congruence between DNA metabarcoding and traditional methods we report here can also be influenced by the overrepresentation of studies conducted in Europe and North America in our data set (Figure 2) where knowledge of fish populations is most advanced. In megadiverse and less studied regions such as the Amazon, DNA metabarcoding and traditional methods can show more contrasting patterns (e.g., Jackman et al., 2021). Finally, fish being not as genetically divergent as plankton, microphytobenthos, and macroinvertebrates, scientists have been able to develop highly specific primers for this group (Kelly et al., 2014; Miya et al., 2015; Valentini et al., 2016) which match well with the diversity of organisms captured and identified using traditional methods.

It is important to note that our conclusions are drawn using one standard measure of diversity, namely richness. Other metrics, like Shannon or Simpson indices that take into account the relative abundance of taxa, could lead to more nuanced conclusions, in particular because the issue of abundance with metabarcoding is yet to be solved (Piñol et al., 2015; Visco et al., 2015). Moreover, we did not investigate how comparable beta diversity (i.e., the species turnover among sites) was between the two methods. This is because beta diversity is more complicated to record and synthesise from multiple sources, as it is often reported using different metrics. This calls for a more standardised way of reporting data enabling future assessments and meta‐analyses. Nonetheless, beta diversity remains an important component of diversity and can give different results if analysed through DNA or traditional methods (Bleijswijk et al., 2020).

Recently, several meta‐analyses have investigated a variety of questions regarding DNA metabarcoding. This includes the diversity of methods used for DNA‐based approaches in ecological assessment using benthic macroinvertebrates (Duarte et al., 2021), the comparison of species detection probability between eDNA and traditional methods (Fediajevaite et al., 2021), or the correlation between species‐specific eDNA concentration and species abundance (Yates et al., 2019). This indicates that the field is gaining maturity and there is now sufficient data available to synthesise and draw general conclusions. It must be noted that this effort is possible only if studies report relevant and extractable data in comparable, complete, and standardised ways. Thus, we stress the importance that future studies comparing traditional methods and DNA metabarcoding provide consistent information about methods and results. As a starting point, we suggest that all variables listed in Table S1 should be systematically reported in an accessible way. Adhering to simple standards will help to improve reproducibility and comparability among studies and facilitate future syntheses (Gerstner et al., 2017).

In conclusion, while DNA metabarcoding has great potential for biodiversity assessment in aquatic ecosystems, we need to consider the implications of significant discrepancies between traditional methods and DNA metabarcoding‐based methods for particular organismal groups. Here, we showed that DNA metabarcoding and traditional methods give similar estimates of taxonomic richness across major organismal groups. This can make these tools interoperable, for example to study patterns and trajectory of biodiversity at large spatial and temporal scales. Importantly, however, while the two approaches still differ on the identity of the species detected, especially in macroinvertebrate and planktonic and microphytobenthic communities, they give similar numbers of total taxa recorded. This may be a problem if the objective is to replace one method with another in long‐term monitoring programs where taxon identity is important. Our results suggest that, for studies targeting fish communities, eDNA metabarcoding is ready to replace invasive methods traditionally used to study the richness and taxonomic composition (species presence‐absence). However, our study did not investigate the capacity of DNA metabarcoding to estimate biomass and species abundance, which are known to be poorly quantified by this method (Lamb et al., 2019), but are often an integral part of ecological monitoring (e.g., for monitoring fish stocks, or for monitoring protected populations). For smaller organisms like macroinvertebrates, plankton, and microphytobenthos, DNA metabarcoding will probably be more a complementary approach, capable of revealing aspects of biodiversity that were previously ignored or underestimated by traditional methods.

## AUTHOR CONTRIBUTIONS

Florian Altermatt, François Keck, and Rosetta C. Blackman conceived the study. All authors collected the data. François Keck analysed the data, François Keck, Rosetta C. Blackman, and Florian Altermatt wrote the manuscript with contributions from all authors.

### OPEN RESEARCH BADGES

This article has earned an Open Data Badge for making publicly available the digitally‐shareable data necessary to reproduce the reported results. The data is available at https://github.com/fkeck/DNA_meta-analysis.

## Supporting information

Supplementary Material

## Data Availability

The data and R scripts to reproduce the analyses and results have been made available at https://github.com/fkeck/DNA_meta‐analysis.
